# Smoking Cessation in Lower Socioeconomic Groups: Adaptation and Pilot Test of a Rolling Group Intervention

**DOI:** 10.1155/2021/8830912

**Published:** 2021-03-08

**Authors:** Lorraine L. Landais, Els C. van Wijk, J. Harting

**Affiliations:** ^1^Amsterdam UMC, University of Amsterdam, Department of Public and Occupational Health, Amsterdam Public Health Institute, Meibergdreef 9, Amsterdam, Netherlands; ^2^Amsterdam UMC, Vrije Universiteit Amsterdam, Department of Public and Occupational Health, Amsterdam Public Health Institute, Van der Boechorststraat 7, Amsterdam, Netherlands

## Abstract

**Background:**

Socioeconomic inequalities in smoking rates persist and tend to increase, as evidence-based smoking cessation programs are insufficiently accessible and appropriate for lower socioeconomic status (SES) smokers to achieve long-term abstinence. Our study is aimed at systematically adapting and pilot testing a smoking cessation intervention for this specific target group.

**Methods:**

First, we conducted a needs assessment, including a literature review and interviews with lower SES smokers and professional stakeholders. Next, we selected candidate interventions for adaptation and decided which components needed to be adopted, adapted, or newly developed. We used Intervention Mapping to select effective methods and practical strategies and to build a coherent smoking cessation program. Finally, we pilot tested the adapted intervention to assess its potential effectiveness and its acceptability for lower SES smokers.

**Results:**

The core of the adapted rolling group intervention was the evidence-based combination of behavioral support and pharmacotherapy. The intervention offered both group and individual support. It was open to smokers, smokers who had quit, and quitters who had relapsed. The professional-led group meetings had a fixed structure. Themes addressed included quitting-related coping skills and health-related and poverty-related issues. Methods applied were role modeling, practical learning, reinforcement, and positive feedback. In the pilot test, half of the 22 lower SES smokers successfully quit smoking. The intervention allowed them to “quit at their own pace” and to continue despite a possible relapse. Participants appraised the opportunities for social comparison and role modeling and the encouraging atmosphere. The trainers were appreciated for their competencies and personal feedback.

**Conclusions:**

Our adapted rolling group intervention for lower SES smokers was potentially effective as well as feasible, suitable, and acceptable for the target group. Further research should determine the intervention's effectiveness. Our detailed report about the adaptation process and resulting intervention may help reveal the mechanisms through which such interventions might operate effectively.

## 1. Introduction

About one-third of the European health inequalities can be explained by socioeconomic inequalities in smoking rates [1]. Despite the efforts of contemporary tobacco control policies to assist smokers in lower socioeconomic status (SES) groups to quit [2], inequalities in smoking rates not just persist, but tend to increase over time [3]. In the Netherlands, for instance, the 2017 smoking prevalence in lower SES groups was 27.1%, compared to 15.6% in higher SES groups [4]. An important explanation for the persistence of inequalities in smoking rates—and thus in health—is that smokers in lower SES groups find it more difficult both to quit and to remain abstinent [5]. Even cessation programs that offer an evidence-based combination of behavioral support and pharmacotherapy, generally considered to be the most effective quit support [6], typically result in lower quit rates for smokers in lower SES groups [7]. Given the lower participation rates and higher dropout and relapse rates for lower SES smokers, such smoking cessation programs are regarded as insufficiently accessible and appropriate for this specific population [8–10].

Therefore, it has been repeatedly recommended to develop smoking cessation interventions that are tailored—or adapted—to the specific needs of lower SES smokers [2, 7, 10, 11]. Although evidence about cessation programs that specifically work for disadvantaged smokers is sparse [10, 12], we are aware of two extensively tailored interventions [13, 14], which showed equity-positive effects [15, 16]. However, a recent review and metaregression found that tailored interventions were as effective as nontailored interventions in bringing about smoking cessation in lower SES groups [11]. This finding led to the conclusion that tailored individual-level smoking cessation approaches do not yet fulfill their expected important aim in reducing smoking-related health inequalities [11]. One explanation may be that lower SES smokers still face more barriers to achieving long-term abstinence than higher SES smokers [12]. Therefore, the challenge of achieving improved long-term smoking cessation in disadvantaged smokers warrants further research into adapting smoking cessation interventions to the needs of lower SES smokers [11, 12], especially in terms of responding to their socioeconomic and social position [10, 17, 18].

The aim of our study was to address this challenge and to systematically adapt and pilot test a smoking cessation intervention for lower SES smokers. Briefly, adapting interventions involves selecting effective core components from existing interventions and tailoring these to the needs of a specific target group [19, 20]. Such adaptations may concern the content, the delivery mode, and the conditions for implementation of an intervention [21, 22]. The present adaptation study was commissioned by the municipal authorities of Amsterdam, the Netherlands. The objective of the commission was to contribute to the municipal poverty policy objective of improving the health of the city's most deprived inhabitants by providing effective smoking cessation support.

In this paper, we report in detail about both the adaptation process and the resulting adapted intervention. In doing so, we want to provide others with the opportunity to take advantage of our experiences and to build on our research findings [14, 23, 24]. If appropriate, others may also want to replicate our adaptation efforts or to implement and evaluate the adapted intervention [25]. Such endeavors may help to identify the active intervention components and the effective methods and applications underlying these components [25, 26]. Therefore, our study may help to elucidate the mechanisms by which smoking cessation interventions for smokers in lower SES groups might operate effectively [12].

## 2. Methods

In the adaptation process (January 2016–June 2017), we followed the usual phases and steps [27, 28]. To specify the methods and activities in the different steps, we used Intervention Mapping for Adaptation (IM Adapt): a protocol for using theoretical, empirical, and practical information to adapt behavioral change interventions in a structured and iterative way [19, 20]. Here, we present a summary of the methods used and activities employed. For an overview and further details, see Supplementary file 1.

**Figure 1:**
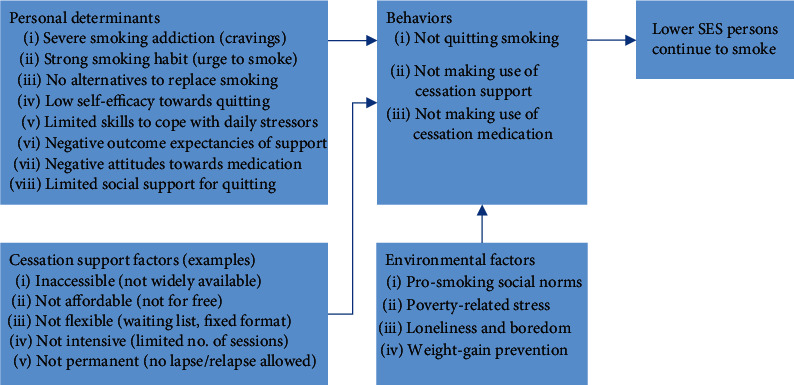
Logic model of factors associated with continued smoking in lower SES persons.

**Figure 2:**
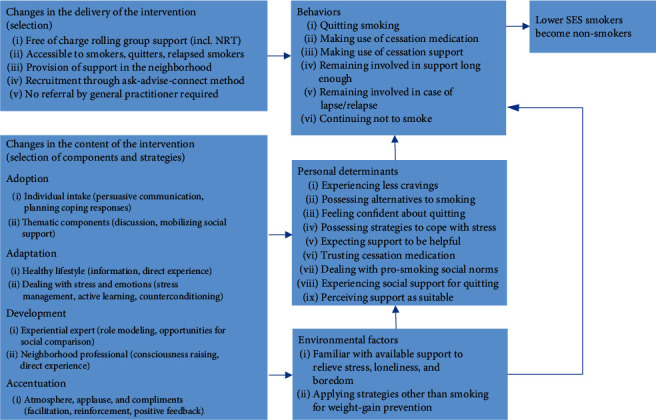
Logic model of change to improve smoking cessation support for lower SES persons.

In the *exploration phase*, we conducted an extensive needs assessment, including a literature review and interviews with both the target group and professional stakeholders. Based on the information collected, the research team developed a logic model of the problem, specifying factors associated with continued smoking by lower SES persons (Figure 1), and a logic model of change, showing how to improve the smoking cessation support for this target group (Figure 2). Next, we reviewed the literature, appraised the recommendations of stakeholders, and searched the national intervention database in order to select candidate interventions for adaptation. A basic requirement was that these interventions should provide an evidence-based combination of pharmacotherapy and behavioral support [6].

In the *preparation phase*, we decided which aspects needed adaptation, using the two logic models that had resulted from the needs assessment. First, we compared the candidate interventions with respect to the behavior change methods they applied and the determinants they addressed. We also reviewed these details for two other interventions tailored to lower SES persons [29, 30]. We then decided which intervention components could be adopted, which needed adaptation, and which should be newly developed. In accordance with IM Adapt [19, 20], we selected effective methods (e.g., modeling) for changing the determinants (e.g., social support and self-efficacy) of health-related behavior (i.e., smoking and quitting). This selection was guided by two taxonomies, one of theory-based behavior change methods [31], the other of behavior change techniques used in individual behavioral smoking cessation support [32]. The research team translated the selected methods into practical strategies (e.g., sharing experiences and role modeling by a lower SES ex-smoker). We then combined these strategies into a coherent smoking cessation program. Other activities included planning the intervention's implementation (e.g., adapting materials and organizing the pilot implementation) and planning the evaluation (e.g., deciding about research methods and developing suitable research instruments).

In the *implementation phase*, the adapted intervention was pilot tested in a one-group pretest-posttest study design. We used recording forms, observations, and interviews with both the participants and the smoking cessation trainers to get an impression of the participation of the target group and the effectiveness of the intervention. With the same methods, we also assessed the intervention's feasibility (Is the intervention being implemented as intended?), suitability (Does the intervention contribute to the changes and benefits expected?), and acceptability (Is the intervention being appreciated by the implementers and the target group?) [33, 34]. The findings of the pilot study were used to further revise the intervention. A description of the final rolling group smoking cessation intervention for lower SES smokers was approved by the Dutch database for certified health promotion interventions (http://www.loketgezondleven.nl/) [35].

According to the Dutch Medical Research Involving Human Subjects Act, this study did not require approval by a medical research ethics committee in the Netherlands.

## 3. Results

First, we present the adapted rolling group intervention for lower SES smokers [35]. In doing so, we follow the recommendation for reporting group-based behavior change interventions [36]. Next, we report, step by step, the findings from the adaptation process, with the intention of making it clear how these findings fed the decisions leading to the final intervention. Finally, we present the core findings of the pilot test, including the rolling group intervention's potential effectiveness, and its feasibility, suitability, and acceptability in the perception of the participants and the trainers [37].

### 3.1. Adapted Rolling Group Intervention

#### 3.1.1. General Approach

To enhance the accessibility for lower SES smokers, the rolling group intervention is provided free of charge. It includes a repetitive series of group meetings led by two experienced smoking cessation trainers skilled in working with the target group. The meetings are open to smokers and ex-smokers, both of whom can take part for as long as they want to. Participants are also permitted to bring a friend or relative. The trainers assist with the use of smoking cessation medication and offer behavioral support. With respect to medication, nicotine replacement therapy (NRT) is offered free of charge at the group meetings, while varenicline can be obtained on prescription, which is mostly reimbursed by the participants' health insurance. Behavioral support comprises both individual components and group meetings. The advisors use a manual that includes instructions for each of the intervention components. The participants receive a workbook with practical exercises to be performed during the group meetings. The language used during the rolling group meetings and for the materials was Dutch (B1-B2 level).

#### 3.1.2. Individual Support

The individual intervention components (Supplementary file 2A) are primarily meant to increase awareness, motivation to quit, social support, and continued participation. All participants get an intake interview of 60 minutes. Depending on individual needs, one or two extra intake sessions can be scheduled. During the intake process, the consequences of smoking (A1, A2), the use of medications (A3, A4), and setting a quit date (A5) are discussed. By way of preparatory homework assignment, participants are asked to keep a smoking diary and fill in a personal quit plan (A6, A7). Further individual support includes brief face-to-face support for new participants at the end of their first group meeting (A8) and brief telephonic support in between group meetings for participants who are new, are still smoking, or have recently quit smoking (A9).

#### 3.1.3. Group Support

One rolling group series includes eleven group meetings, lasting 90 minutes each. Each of these weekly meetings has the same structure (Supplementary file 2B), but addresses a different theme (Supplementary file 2C).

*(1) Group Meeting Structure*. Each meeting starts with cheerful music to create a positive atmosphere (B1). This is followed by CO measurements to provide feedback on health gains, so as to reinforce the motivation to quit (B2). Next, to further increase and consolidate the motivation to quit, they rate the importance of quitting, their confidence in quitting, and the cravings they have experienced (B3). This brief survey in the participants' workbook serves as preparation and input for the group conversation, during which they exchange quit experiences to boost their self-efficacy (B4). Meanwhile, they are encouraged to give each other positive feedback so as to enhance social support. Halfway through the meeting, an “energizer” can be offered to keep the participants engaged (B5). The next time slot is dedicated to the central theme of the meeting (B6; these themes are further explained below). Each meeting concludes with a visualization exercise meant to help participants cope with stress (B7). After the meeting, participants using NRT are given enough medication for the next week (B8). To maximize participation, participants receive free public transport tickets (B9) and a stamp on a card—adding up to a certificate—as a reward for their presence (B10).

*(2) Group Meeting Themes*. The aims of the central theme of the meetings vary, but often include enhancing self-efficacy and strengthening coping strategies. “The balance” (C1) addresses the health gains of smoking cessation by weighing pros and cons of smoking and quitting. Four meetings concentrate on quitting-related coping skills: coping with emotions (C3), pitfalls (C6), negative thoughts (C8), and withdrawal symptoms (C10). In these meetings, an “experiential expert” serves as a role model by explaining how he himself or she herself has dealt with these challenges. Five meetings address other health-related or poverty-related themes: adopting a healthy diet (C2), finding meaningful daytime activities (C4), engaging in physical activity (C5), managing one's finances (C9), and managing stress (C11). In these meetings, a “neighborhood professional” presents a locally available theme-related activity or service, which participants can immediately get acquainted with through a simple game or a short exercise. “Keep it up!” (C7) supports both knowledge about smoking, with a quiz led by a pulmonologist, and relapse prevention, with a scenario-based exercise for planning coping responses ahead.

### 3.2. Adaptation Process

#### 3.2.1. Exploration Phase

*(1) Step 1: Needs Assessment*. Overall (see Figure 1), our literature search revealed that lower SES smokers find it more difficult to quit because of severe nicotine addiction [9, 10], strong smoking habits, and the absence of alternative behaviors that can replace smoking [9, 38, 39]. Also, they experience low self-efficacy and coping skills [10, 40], low social support for quit attempts [10, 29, 39, 41], and a strong prosmoking social norm [38, 42]. In addition, lower SES smokers are often unfamiliar with the services available [43, 44] and tend to have negative attitudes towards smoking cessation support (e.g., perceiving support as ineffective and expensive) [9, 43, 45]. Important reasons to smoke include coping with poverty-related stress [10, 38], loneliness and boredom [9, 42], and weight-gain prevention [39]. Additional needs indicated by lower SES smokers included peer support [10, 29, 41] and support regarding material and social circumstances [42, 46–48].

The importance of these findings was confirmed in the interviews with both lower SES smokers and experts/stakeholders. As an additional need, the target group mentioned support for adopting a healthy lifestyle in general (e.g., diet and exercise). The experts/stakeholders agreed that smoking cessation support should be adapted to the target group's specific needs, implying that the support should be easily accessible (e.g., offered in their own neighborhood), affordable (i.e., cheap or for free), flexible (e.g., allowing for relapses), intensive (e.g., extra support in between formal meetings), and permanent (i.e., allowing for long-lasting and repeated participation) [9, 40, 41, 43, 45, 49].

Based on the above needs, the program goal we formulated for the adapted intervention was to transform lower SES smokers into nonsmokers (see Figure 2). Performance objectives encompassed quitting smoking, participating in the program, and staying involved in the program as long as necessary to continue abstinence. Personal determinants included alternatives for smoking, coping skills, and support experienced. The most important environmental factor was familiarity with support in other health and social domains.

*(2) Step 2: Intervention Selection*. Neither our literature search nor the Dutch database for certified interventions provided examples of smoking cessation services that met the above requirements, especially in relation to the lower SES smokers' deprived social position. The literature showed that multisession, face-to-face behavioral support is able to increase quit success and is affordable in middle- and high-income countries, such as the Netherlands [50]. Although the evidence is mixed [51], low-income smokers may also benefit more from group support compared to individual support [47, 49], as groups may create opportunities to share experiences and to give and receive social support. The only group intervention adapted to lower SES smokers we identified did improve short-term outcomes (e.g., days to relapse), but not long-term outcomes (3-month and 6-month abstinence), for which extended tailored support should be provided [15].

An example of such extended support is a rolling group intervention, without fixed start or end dates, which allows smokers to enroll at any time, to take part as long as they want, and to reenroll in case of relapse [52]. Such rolling group support may be more successful than conventional cessation support in reaching lower SES smokers [53] and in helping these smokers to quit [54]. However, rolling group support does not always outperform conventional support with respect to quitting [53], which may be explained by high dropout rates and the relatively limited attention paid to the target group's specific needs [52].

Most lower SES smokers we interviewed were positive about the rolling group concept. However, the professional stakeholders we talked to pointed out that group support in the Netherlands is scarce and—in their opinion—not or insufficiently “rolling.” Alternatively, they recommended a certified Dutch closed group intervention tailored to smokers with a psychiatric background, as the needs it addressed were in part comparable to those of lower SES smokers [55]. Finally, we opted for (1) the rolling group intervention as the core intervention for us to adapt, as its delivery mode already addressed multiple needs of lower SES smokers, and (2) the Dutch closed group intervention to support the adaptation process, as its content was already partly tailored to lower SES smokers' needs.

#### 3.2.2. Preparation Phase

*(1) Step 3: Deciding What Needs Adaptation*. To decide what needed adaptation, we examined the content and delivery mode of both the rolling group and the closed group intervention. We assessed in detail the “fits and mismatches” in the themes dealt with, the behavior change methods used, and the determinants addressed. We based this assessment on a detailed report of the rolling group support intervention and on the extensive trainers' manual and participants' workbook of the Dutch closed group intervention [52, 55]. In search of ways to solve any mismatches identified, we also made use of other sources. These included the two taxonomies of behavior change methods and techniques referred to above [31, 32] and two Dutch behavior change interventions, one tailored to lower SES smokers [29], the other to help lower SES persons self-manage their diabetes [30]. Decisions about adaptations following from this assessment are illustrated in the next step.

*(2) Step 4: Making Adaptations*. We first adopted existing components that were already tailored to the needs of lower SES smokers, then secondly adapted those components that were suitable for the target group but needed further tailoring, and thirdly developed new components for those determinants that were not yet sufficiently addressed by the adopted and adapted components.

For example, from the rolling group intervention, we *adopted* the accessibility for both nonsmokers and smokers (e.g., allowing for relapses and repeated quit attempts) and the absence of an end date for participation (to offer long-lasting support). From both example interventions, we used the group conversation and the fixed moment for paying compliments (e.g., to increase social support and self-efficacy). From the closed group intervention, we adopted the individual intake (e.g., to stimulate awareness and set a quit date) and several thematic components (e.g., weighing pros and cons of smoking and quitting and practicing coping strategies in case of withdrawal symptoms).

Components we *adapted* included the use of nicotine replacement therapy (by discussing it in more detail and by offering it for free) and components of some other themes, such as a healthy diet, physical activity, and coping with stress (e.g., by adding exercises that included practical learning and direct experience).

Components we *newly developed* included the provision of additional individual support, such as before and in between the group sessions. Other such components addressed financial issues, activities to replace smoking (to break the smoking habit), and ways to counteract loneliness and boredom (to prevent relapses). Also new were the involvement of an experiential expert, i.e., a lower SES ex-smoker serving as a role model (e.g., to increase social support and self-efficacy), and a neighborhood professional who provided a practical introduction to nearby services (e.g., a debt counselor introducing financial help) or activities (e.g., a trainer of relaxing activities introducing yoga) in relation to the theme of the meeting.

A final example of tailoring was the emphasis, throughout the entire program, on reinforcement and positive feedback.

For a complete overview of adopted, adapted, and newly developed components, see Supplementary files 2A–2C.

*(3) Step 5: Planning the Implementation*. To prepare for the pilot implementation of the adapted intervention, the research team adjusted trainers' manual and the participants' workbook from the Dutch closed group intervention. Furthermore, we contracted and instructed trainers, selected a suitable setting in the neighborhood, and recruited and briefed both experiential experts and neighborhood professionals. Finally, we engaged and trained social workers, i.e., client managers of the municipal Department for Social Security and Participation and debt counselors of a local social work organization, to recruit lower SES smokers into the intervention using the ask-advise-connect method [56].

*(4) Step 6: Planning the Evaluation*. The aim of the pilot test was twofold. First, we wanted to know which lower SES smokers would participate in the interventions, as well as their attendance, dropout rates, and smoking cessation rates. For this purpose, we developed an intake form, to be completed by the participants, and two recording forms, to be completed by the trainers. Second, we aimed to examine feasibility, suitability, and acceptability of the adapted rolling group intervention. To this end, we designed (i) a visual observation template to monitor the implementation of intervention components; (ii) an observation scheme to make notes about the atmosphere, the interactions between the attendees, and the responses of the participants to the different intervention components; and (iii) interview guides to examine the views and experiences of both the participants and the trainers.

#### 3.2.3. Implementation Phase

*(1) Step 7: Pilot Testing*.

- *Setting and Recruitment*. The rolling group intervention was pilot tested in a community center in a deprived neighborhood in Amsterdam. The pilot test included two series of 11 group meetings, which were led by two experienced smoking cessation trainers. Lower SES smokers who were motivated to quit were invited to take part by the social workers by using proactive ask-advise-connect recruitment [56].
- *Data Collection and Analysis*. The intake form was completed by 21 participants (95%). The recording forms were completed by the trainers for all of the 22 participants. The first 15 meetings were observed in full, the last seven meetings only in full if they had been substantially revised (see Step 2: Intervention Revision). Interviews with the participants were held after 12 meetings (*n* = 13; half of which smoked). The trainers were formally interviewed after six and 17 meetings, while informal debriefings were held after each of the first 11 group meetings. The quantitative data were summarized in Excel; the qualitative data were manually analyzed [57].

*(2) Step 8: Intervention Revision*. After six rolling group meetings, the trainers regarded the intervention as insufficiently “rolling,” as some elements still assumed that all participants were in the same stage of the cessation process. Therefore, we changed the order of the meetings and relocated—mostly personal—elements to the individual intake interview and homework assignment.

This change also responded to the trainers' perception that participants, on top of the group meetings, needed more personal attention, specifically to facilitate the early stages of the cessation process. Therefore, we extended the individual intake interview, allowed for up to three intake interviews per participant, and encouraged the trainers to phone participants in between the group meetings, especially those who recently had made a quit attempt.

More personal attention was also expected to result in the presence of more nonsmokers than smokers during the group meetings, as such a ratio was desirable according to the trainers. To meet this condition, we also introduced clearer rules of play, such as having the intention to quit rather than just wanting to cut down smoking, which had to be evident from setting a quit date.

Based on the recording form and the trainers' suggestions, we added two themes, i.e., coping with emotions and dealing with pitfalls. Similarly, we added some strategies to prevent participants from dropping out of the rolling group (e.g., small presents that provide people with activities as an alternative to smoking). We also followed the participants' advice to set quality criteria for both the experiential expert (e.g., having quit smoking for at least a year) and the neighborhood professional (e.g., being able to connect with and enthuse the participants).

Since participants felt uncomfortable with the fixed moment for paying compliments, we instead asked the trainers to encourage participants throughout the meetings to give each other positive feedback. As some of the meetings included too many components, we removed those elements that appeared least suitable for either a rolling group (i.e., finding a buddy within the group) or the target group (e.g., doing homework assignments in between group meetings).

Finally, we asked the trainers to deal more effectively with language barriers by further simplifying their language use and by discussing the workbook exercises in the whole group. Although further tailoring to language barriers might be required, this was not possible during the pilot study due to time constraints.

*(3) Step 9: Evaluation Findings*.

**Figure 3:**
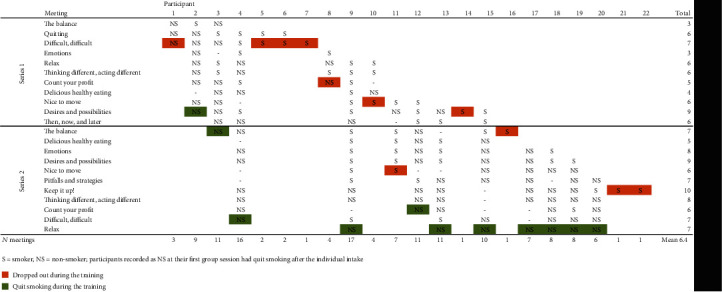
Attendance and smoking status of participants per rolling group meeting (January–June 2017).

- *Participants*. In total, 22 lower SES smokers received an intake interview and participated in the rolling group. Their mean age was 52.6 years and 59% were female. The participants represented eight different ethnic backgrounds: Dutch (*n* = 5), Turkish (*n* = 5), Moroccan (*n* = 3), Surinamese (*n* = 3), Indonesian (*n* = 2), Ghanaian (*n* = 1), German (*n* = 1), and English (*n* = 1). None of the participants had a paid job. Most were on social security (*n* = 12), unemployed (*n* = 3), or disabled (*n* = 2). Most (*n* = 18) experienced difficulties to get by from their income. The mean number of cigarettes smoked at intake was 22.9.
- *Participation, Dropout, and Smoking Cessation*. The 22 participants attended an average of 6.4 group meetings (range 1–17) (Figure 3). Half of the participants successfully quit smoking, although most of them relapsed into smoking at least once. All successful quitters used pharmaceutical support. Four of them left the rolling group with a certificate, while seven preferred to participate to the end of the pilot test. For these stayers, the trainers organized—at their own initiative—monthly follow-up meetings. Of the eleven participants who did not successfully stop smoking, three appreciated the rolling group intervention, but could not continue their participation due to personal circumstances. Two other participants left the group because of a disagreement with the trainers (about the distribution of NRT medication) and one did not feel he fitted in with the group. Five participants did not return for unknown reasons.
- *Feasibility, Suitability, and Acceptability*. Both participants and trainers were very enthusiastic about the training program. The participants appreciated the rolling group concept, as it allowed them to “quit at their own pace” and to continue to participate despite a possible relapse. They found the atmosphere encouraging, due to the positive feedback and humor from both participants and trainers. The participants actively took part in the exercises and discussions. They were open about their successes and failures, gave each other advice, and offered social and emotional support. Participants found the group conversations to be the most helpful to quit smoking, as they enabled social comparisons (experiencing comparable problems) and role modeling (learning how to cope with these problems). They appreciated the trainers for their knowledge (e.g., on smoking-related issues), competencies (e.g., their understanding and positive and activating approach), and personal feedback. Participants perceived the experiential experts (showing them that success is possible) and the neighborhood professionals (introducing them to new activities and support) also to be important for quitting smoking. They reported that taking part in the training program had been facilitated by the opportunity to get acquainted with the intervention as well as the free of charge support and NRT. They acknowledged that continued participation was encouraged by the small presents offered as well as by the stamps and the certificates they received for their attendance. Observations revealed that the meetings were sometimes somewhat chaotic, e.g., if participants entered late or took a short break, but for the experienced trainers, this was not a problem. The trainers were also confident about managing groups of up to eight participants on their own. Due to time constraints, mostly some elements of the meetings were left out. Finally, the trainers advocated the continuation of the rolling group, as this type of smoking cessation support would be of added value for lower SES smokers wanting to quit.

## 4. Discussion

### 4.1. Summary

We systematically adapted a smoking cessation intervention to the needs of lower SES smokers. This resulted in a tailored rolling group intervention. In a pilot test, recording forms showed that half of the participants in the intervention quit smoking. In the accompanying process evaluation, observations and interviews made clear that the rolling group intervention was largely feasible, suitable, and acceptable.

### 4.2. Reflections

Based on our one-group pretest-posttest pilot study, it is difficult to reliably interpret the finding that 50% of the participants quit during the pilot implementation of the tailored rolling group intervention. In any case, this 50% falls within the range of abstinence rates reported for other smoking cessation interventions adapted to or developed for lower SES smokers [15, 16]. For instance, a closed group intervention reached initial and 3-month abstinence rates of 87.7% and 48.2%, respectively [15], and an individual internet-based intervention achieved a 6-month abstinence rate of 8% for the participating lower SES smokers [16]. Both these interventions also yielded equity-positive effects. Hence, the adapted rolling group intervention may be a promising alternative way to support lower SES smokers in quitting. Providing a variety of such alternatives could be important in response to the different needs for support expressed by lower SES smokers wanting to quit [58].

Our process evaluation endorsed the suitability of the adapted rolling group intervention, meaning that the intervention may indeed have contributed to the changes and benefits found [33]. Although it has been argued that in adaptation processes the function of intervention elements should be prioritized over the form of the intervention [59], our pilot study suggests that both aspects may be just as important for the effectiveness of an intervention. For instance, both the interviews and the recording forms in our pilot study indicated that the highly flexible rolling group format and the encouraging atmosphere during the group meetings were both crucial in keeping participants involved in the intervention for long enough to allow them to quit smoking at their own pace. By providing insights like these, our study confirms the importance of systematically adapting smoking cessation interventions [19] and of reporting in detail the adaptation process [21], the adapted theory-based intervention [60], and the pilot test results [26]. Therefore, studies like ours may contribute to the required understanding of the adaptations necessary to design interventions that can achieve equity-positive effects [12].

### 4.3. Limitations

The first limitation is that our pilot study had limited internal and external validity. Our quasi-experimental design did not allow us to assess the intervention's effectiveness, and financial and time restraints precluded us from assessing the participants' smoking status on the longer term. The participants in our study were mostly older people. From this sample, it is difficult to infer the intervention's potential effectiveness and acceptability for lower SES smoker of a younger age.

The second limitation is that we were not able to properly evaluate the recruitment of participants for the rolling group intervention. The ask-advise-connect recruitment [56] by social workers that we organized resulted in a relatively small number of lower SES smokers in the intervention. Therefore, a real-world implementation of the adapted rolling group intervention may require stronger proactive outreach methods and/or *word of mouth referral* by the target group itself [61, 62]. Due to the small scale and the short duration of the pilot test, we were also unable to assess the role of the trainer's competencies in assisting participants to quit and to make an analysis of agreement between the trainers.

The third limitation is that we adapted a group-based intervention by applying methods aimed at changing determinants of health behavior [31], rather than methods to facilitate group processes [63]. Although our intervention paid considerable attention to social factors (e.g., social support, social engagement, and social comparison), it could probably benefit from applying additional methods to strengthen group interaction, organization, and leadership [63].

The final limitation is that, although we took into account the socioeconomic and social position of the participants, we were hardly able to address these social factors as causal determinants of smoking. Despite including intervention components aimed at coping with the stress that often accompanies a lower SES position [10] and giving participants the opportunity to get acquainted with support in the socioeconomic and social domains, a more profound approach may be needed to substantially lessen the social gradient in smoking rates [11].

### 4.4. Conclusions

Our systematic approach resulted in a smoking cessation rolling group intervention adapted to the needs of lower SES smokers. From our pilot study, we conclude that the effectiveness and the feasibility, suitability, and acceptability of the intervention are promising. Further research should determine the effectiveness of the rolling group intervention. Our detailed report about the adaptation process and the resulting intervention may help to reveal the mechanisms by which such interventions might operate effectively.

## Data Availability

The quantitative data used to support the findings of this study are included within the article. Dutch summaries of the qualitative data are available from the corresponding author upon request.

## Supplementary Materials

Supplementary Materials Supplementary file 1 provides an overview of methods and activities employed in each phase of the intervention adaptation process. For the resulting smoking cessation rolling group intervention, Supplementary file 2 further specifies the individual components (file 2A), the structure of the group meetings (file 2B), and the content of the group meetings (file 2C).
