# Supplementary material for: Smoking Cessation in Lower Socioeconomic Groups: Adaptation and Pilot Test of a Rolling Group Intervention
Source: Biomed Res Int. 2021 Mar 8;2021:8830912. doi: 10.1155/2021/8830912 (PMC7963897; doi:10.1155/2021/8830912)
Supplement: Supplementary Materials — Supplementary file 1 provides an overview of methods and activities employed in each phase of the intervention adaptation process. For the resulting smoking cessation rolling group intervention, Supplementary file 2 further specifies the individual components (file 2A), the structure of the group meetings (file 2B), and the content of the group meetings (file 2C). [file 8830912.f1.zip › 8830912.f1/Suppl 1 Adaptation process FEB 2021 BIOMED RES INT DEF.docx]

**Supplementary file 1.**

**Overview of methods and activities for each phase of the adaptation process** [1-4]

| **Exploration phase (January-June 2016)** | |
| --- | --- |
| **1. Needs assessment** | |
| - 1. Literature review | - Literature review [5, 6] on specific needs of lower SES smokers and barriers to the uptake of support. - Databases: PubMed, Cochrane, Web of Science, Google Scholar. - Selection of 77 papers, including reviews, experimental studies, observational studies. - Thematic analysis using a framework approach,[7] resulting in information on: determinants of smoking and smoking cessation; recruitment strategies; smoking cessation interventions. |
| - 1. Interviews with target group | - Eight focus groups and nine individual semi-structured interviews [8] with lower SES smokers (n=32) and ex-smokers (n=19), recruited via key informants, purposive and snowball sampling (verbal informed consent). - Mean age 52 years (SD=9.5); 50% female; ethnic background mainly Dutch, Turkish or Surinamese, reflecting the Amsterdam population with highest smoking prevalence. - Computer assisted framework analysis [7, 9] of interview transcripts on the themes of: smoking and smoking cessation, determinants of smoking, quitting and relapsing; recruitment strategies; smoking cessation support. |
| - 1. Interviews with stakeholders/experts | - Semi-structured interviews [8] with 45 experts and/or stakeholders, alone or in couples, including smoking cessation trainers, service providers, developers and researchers of cessation support, and policy officers from municipality and insurance company. - Detailed minutes of topics discussed: available smoking cessation support, appropriateness of support for target group, and, if applicable, personal experiences with providing such services. - Thematic analysis [7] resulting in recommendations for: context, form and content of cessation support; informing, recruiting and motivating lower SES smokers/intermediaries; necessary organizational and policy support. |
| - 1. Activities of the research team | - Developing logic model of the problem, specifying factors associated with continued smoking by lower SES persons (see Figure 1A). - Developing logic model of change, showing how to improve the smoking cessation support for lower SES persons (see Figure 1B). - Writing program goals: transforming lower SES smokers into non-smokers, by quitting smoking, participating in the program, and staying involved as long as necessary to continue abstinence (based on Figure 1B). |
| 1. **Intervention selection** | |
| - 1. Activities of the research team | - Reviewing the literature on smoking cessation interventions for lower SES smokers (see Needs assessment). - Reviewing the recommendations from experts and stakeholders (see Needs assessment). - Searching the national intervention database (www.loketgezondleven.nl) for interventions tailored to lower SES smokers. - Selecting a rolling group smoking cessation intervention tailored to lower SES smokers, which was to be adapted. - Selecting a closed-group intervention tailored to smokers with a psychiatric background to support the adaptation process. |
| **Preparation phase (July 2016-January 2017)** | |
| 1. **Deciding what needs adaptation** | |
| - 1. Activities of the research team | - Selecting a combination of pharmacotherapy and behavioral support as the evidence-based core elements of effective smoking cessation interventions. - Comparing detailed descriptions of selected smoking cessation interventions (i.e. behavior change methods and determinants addressed) with outcomes of needs assessment to assess fits and mismatches. - Reviewing descriptions of other interventions tailored to lower SES persons so as to select appropriate components, theoretical methods and practical applications. |
| 1. **Making adaptations** | |
| 4.1. Activities of the research team | - Adoption of existing intervention components already tailored to needs of lower SES smokers. - Adaptation of intervention components suitable for lower SES smokers but requiring further tailoring. - Development of new intervention components for determinants that were insufficiently addressed by adopted and adapted components. |
| 1. **Planning the implementation** | |
| - 1. Activities of the research team | - Adaptation of trainers’ manual: explaining the rolling group concept, paying attention to the role of experiential experts and neighborhood professionals, and providing detailed instructions for the individual intake interviews, group meetings and support in between meetings. - Adaptation of participants’ workbook: aligning it the adapted and newly developed components, changing assignments in accordance with active and practice-based learning, and simplifying the language. - Organizing pilot implementation of rolling group intervention: two cycles of eleven meetings; recruitment of trainers; selection of community center in deprived neighborhood; recruitment and briefing of “experiential experts” and “neighborhood professionals” (i.e. a dietician or nutritionist, an employee of a community center, a teacher of a physical activity class, a pulmonologist, a debt counsellor, a trainer of a relaxing activity). - Organizing recruitment of participants: instruction and motivation of client managers (department of social security) and debt counselors (social work organization) to recruit participants among their smoking clients. |
| 1. **Planning the evaluation** | |
| - 1. Activities of the research team | Aim: to assess participation, drop-out and smoking cessation in the pilot test   - Development of intake forms for participants (e.g., demographics of participants; including their working situation (e.g., having paid work, receiving a benefit, or following education) and difficulties with getting by from their income)/ - Development of recording forms (e.g., participants’ presence, smoking status, use of medication) to be completed by the trainers.   Aim: to examine the feasibility, suitability and acceptability of the intervention   - Design of visual observation template [8], based on the logic model of change (Figure 1B), the trainers’ manual, and the content and structure of each of the rolling group meetings (see Supplementary files 1-3). - Design of an observation scheme [8], including topics like: atmosphere, interactions (between trainers, between participants and between trainers and participants), and participants’ responses to intervention components. - Development of interview guides [8] for participants and trainers about the feasibility, suitability, and acceptability of the intervention [10]. |
| **Implementation phase (January 2017-June 2017)** | |
| 1. **Pilot testing** | |
| - 1. Setting | The adapted rolling group intervention was pilot-tested in a community center in a deprived neighborhood. Lower SES smokers from that neighborhood were invited to take part by social workers. |
| - 1. Participants | - In total, 22 lower SES smokers participated. - Demographics available for 21 participants. - The mean age was 52.6 years (range: 30 to 64 years); 13 were female (59%). - The ethnic background of the participants was Dutch (n=5), Turkish (n=5), Moroccan (n=3), Surinamese (n=3), Indonesian (n=2), Ghanaian (n=1), German (n=1) or English (n=1). - Work and income: social security (n=12), unemployed (n=3), disabled (n=2), other (n=4); most had difficulties to get by with their income. - At the moment of intake, participants smoked an average of 22.9 cigarettes a day (range: 7 to 38 cigarettes). |
| - 1. Data collection and data analysis | Recording   - Intake forms were completed for all 22 participants by the trainers. - Recording forms were completed by the trainers for all 22 meetings.   Observations   - The first 15 of the 22 meetings were observed in full by one of the researchers. - The last 7 meetings were observed if additional adaptations had been made.   Interviews   - Participants were interviewed after 12 meetings, in one group interview and five telephonic interviews (verbal informed consent). Of the 13 respondents, 6 were still participating in the intervention, 2 had completed the intervention, 5 had dropped out. Half of the respondents smoked at the time of the interview. - Trainers were formally interviewed after six and 17 meetings (verbal informed consent). In addition, informal debriefings were held after each of the 11 meetings of the first cycle of the rolling group. Detailed minutes of all debriefings and interviews were made by the researchers.   Analysis   - Quantitative data were entered and summarized in Excel (descriptives). - Qualitative data (i.e. the detailed minutes of the interviews) were manually analyzed using a thematic approach [7]. |
| 1. **Intervention revision** | |
| 8.1 Activities of the research team | - Changing the order of meetings to make the intervention more ‘rolling’. - Allowing for more intake interviews per participant (more personal attention). - Adding extra elements on dealing with emotions/pitfalls (relapse prevention). - Setting quality criteria for experiential experts and neighborhood experts. - Limiting number of elements per meeting and addressing language barriers. |
| **9. Evaluation findings** | |
| 9.1 Activities of the research team | - Writing an evaluation report for the commissioning agency and stakeholders involved [11]. - Drafting the present manuscript in order to enable further dissemination. - Drafting a description of the intervention in order to enable implementation. |
| **10. Final intervention** | |
| 10.1 Activities of the research team | - Finalizing the intervention description and submitting the intervention to the national intervention database ([www.loketgezondleven.nl](http://www.loketgezondleven.nl)) [12]. |

**References**

1. Movsisyan, A., et al., *Adapting evidence-informed complex population health interventions for new contexts: a systematic review of guidance.* Implement Sci, 2019. **14**(1): p. 105.

2. Escoffery, C., et al., *A scoping study of frameworks for adapting public health evidence-based interventions.* Transl Behav Med, 2018. **9**(1): p. 1-10.

3. Bartholomew Eldredge, L.K., et al., eds. *Planning health promotion programs. An intervention mapping approach*. 4th ed. 2016, Jossey-Bass: San Francisco.

4. Highfield, L., et al., *Intervention Mapping to Adapt Evidence-Based Interventions for Use in Practice: Increasing Mammography among African American Women.* Biomed Res Int, 2015.

5. Greenhalgh, T. and R. Peacock, *Effectiveness and efficiency of search methods in systematic reviews of complex evidence: audit of primary sources.* BMJ, 2005. **331**(7524): p. 1064-5.

6. Green, J. and N. Thorogood, *Qualitative methods for health research*. 3rd ed. 2014, London: Sage.

7. Srivastava, A. and S.B. Thomson, *Framework analysis: a qualitative methodology for applied policy research.* Journal of Administration and Governance, 2009. **4**: p. 12-79.

8. Polit, D.F. and C.T. Beck, *Nursing research: Principles and methods*. 7th ed. 2004, Philadelphia: Lippincott Williams & Wilkins.

9. MaxQDA, *MAXQDA Plus*. 2016, VERBI Software GmbH: Berlin. p. Software for Qualitative Data Analysis.

10. Linnan, L. and A. Steckler, *Process evaluation for public health interventions and research: An overview*, in *Process evaluation for public health interventions and research*, A. Steckler and L. Linnan, Editors. 2002, Jossey-Bass: San Francisco. p. 1-23.

11. Harting, J., L. Landais, and E. Van Wijk, *Smoking cesssation for low-income inhabitants of Amsterdam: Summary report, needs assessment and pilot-test [Dutch]*. 2017, Public Health, University of Amsterdam: Amsterdam.

12. Harting, J., L. Landais, and E. Van Wijk, *Feel Free! Rolling group Smoking cessation support: Description of the Intervention [Dutch]*. 2018, Public Health, University of Amsterdam: Amsterdam.
