# Supplementary material for: Smoking Cessation in Lower Socioeconomic Groups: Adaptation and Pilot Test of a Rolling Group Intervention
Source: Biomed Res Int. 2021 Mar 8;2021:8830912. doi: 10.1155/2021/8830912 (PMC7963897; doi:10.1155/2021/8830912)
Supplement: Supplementary Materials — Supplementary file 1 provides an overview of methods and activities employed in each phase of the intervention adaptation process. For the resulting smoking cessation rolling group intervention, Supplementary file 2 further specifies the individual components (file 2A), the structure of the group meetings (file 2B), and the content of the group meetings (file 2C). [file 8830912.f1.zip › 8830912.f1/Suppl 2ABC JUNE 2020 BIOMED RES INT.doc]

**Supplementary file 2A. Smoking cessation rolling group intervention: Content of the individual components**

| **No.** | **Intervention component** | **Existing (E)**  **Adapted (A)**  **New (N)** | **Objective(s)** | **Theoretical method(s)** | **Practical application(s)** |
| --- | --- | --- | --- | --- | --- |
| *Individual intake* | |  |  |  |  |
| A1. | Discuss health risks of smoking | **E** | - Increase awareness of harmfulness of smoking | - Personalize risk - Consciousness raising | The trainer provides information about the health risks of smoking. |
| A2. | Discuss advantages of smoking cessation | **E** | - Create positive attitude towards smoking cessation | - Belief selection - Persuasive communication | The trainer discusses the advantages of smoking cessation with the participant and uses arguments to convince him. |
| A3. | Information about nicotine addiction | **E** | - Enhance knowledge about nicotine addiction | - Provide information | The trainer explains the ‘nicotine addiction circle’: smoking increases levels of nicotine in the blood; the body breaks down the nicotine, which leads to withdrawal symptoms that cause craving for nicotine and an urge to smoke. |
| A4. | Discuss use of effective medications | **A** | - Create awareness of advantages of medications - Increase motivation to use medications | - Persuasive communication - Provide instruction | The trainer stresses the benefits of effective medications (NRT and other medications) and discusses whether the participant wants to use it. If so, the trainer gives instructions about the use. |
| A5. | Quit date | **E** | - Stimulate participant to set quit date | - Implementation intention - Goal setting | The trainer stimulates the participant to write down his decision to quit smoking and to set a quit date. |
| *Homework assignment* | |  |  |  |  |
| A6. | Smoking diary | **E** | - Create awareness of own smoking behavior | - Self-monitoring of behavior - Active learning | The participant keeps a 'smoking diary' during a week, in which he indicates on a daily basis when, where, why and with whom he smokes.  The participant answers questions about situations in which he smokes and what he learned from previous quit attempts. |
| A7. | Quit plan | **E** | - Prepare for quit day | - Goal setting - Stimulus control | The participant indicates what strategies he will use to prepare himself for the quit day (e.g.: removing cues for smoking and starting the use of medications). |
|  |  | **E** | - Coping with cravings | - Planning coping responses | The participant fills in questions about what he will think or do when he has an urge to smoke. The trainer presents the 'traffic light' scheme as a strategy to cope (Red: 'Count to 10', Orange: 'Think about what to do', Green: 'React'). |
|  |  | **E** | - Identify social support | - Mobilize social support | The participant is asked to write down who will help him when he quits smoking and how these persons can help him. |
|  |  | **E** | - Relapse prevention | - Planning coping responses | The participant indicates high-risk situations and identifies ways to prevent himself from relapse. |
|  |  | **E** | - Strengthen motivation to quit smoking | - Reinforcement - Early commitment | The participant fills in how he will reward himself, e.g. by planning how he will spend the money saved by quitting smoking. |
| *Individual follow-up support* | |  |  |  |  |
| A8. | Individual support for new participants after first group meeting | **N** | - Increase social support - Increase knowledge about medications and the use of it | - Social support - Provide instructions | The trainer gives individual support to participants who joined the rolling group for the first time. The trainer discusses the homework assignments from the intake and gives extra instructions about the use of medications (if applicable). |
| A9. | Telephone support in between group meetings | **N** | - Increase social support | - Social support - Individualization | The trainer calls participants who still smoke and participants who recently quit to provide additional support. Participants also have the opportunity to ask questions. |

**Supplementary file 2B. Smoking cessation rolling group intervention: Structure of the group meetings**

| **No.** | **Intervention component** | **Existing (E)**  **Adapted (A)**  **New (N)** | **Objective(s)** | **Theoretical method(s)** | **Practical application(s)** |
| --- | --- | --- | --- | --- | --- |
| B1. | Music | **E** | - Create a positive atmosphere - Increase engagement | - Facilitation | Pleasant music is played at the start of each meeting. |
| B2. | CO measurement | **E** | - Increase motivation to quit smoking and persevere | - Feedback on performance - Reinforcement | The trainer measures the carbon monoxide (CO) level of participants with a CO meter. This instrument provides participants with direct feedback about their health gain. |
| B3. | Questions about importance of smoking cessation, confidence and cravings | **E** | - Raise consciousness about smoking behavior - Increase motivation to quit smoking | - Self-monitoring of behavior | The trainer asks participants to indicate to what extend (1) they think smoking cessation is important, (2) they feel confident about quitting and (3) they feel an urge to smoke. This information serves as input for the subsequent group conversation. |
| B4. | Group conversation: sharing and discussing experiences | **E** | - Increase social support - Raise awareness about smoking behavior - Increase self-efficacy | - Mobilize social support - Modelling | Every participant is encouraged to share his experiences with difficult moments in smoking cessation with the group. The trainer asks further questions and stimulates participants to give each other positive feedback. |
| B5. | Energizer (optional) | **N** | - Generate energy in the group - Create a positive atmosphere - Increase engagement | - Facilitation | Short breaks during the meeting in which the participants play an easy and funny physical activity game, e.g.: participants stand in a circle and try to keep one or multiple balloons up in the air. |
| B6. | Theme | **A** | - Offer support in different areas that are important for smoking cessation (e.g.: coping with withdrawal symptoms, healthy lifestyle and coping with stress) | - Provide information - Discussion - Direct experience - Modelling | Each meeting includes a different theme (see theme 1‑11 in Table 3). The trainer discusses this theme with the participants. Either a “neighborhood professional” is invited to make participants acquainted with theme-related local services, or an experiential expert is present to share his experiences in relation to the theme. |
| B7. | Visualization | **E** | - Coping with stress - Create positive attitude towards quitting smoking | - Stress management - Self-re-evaluation | At the end of the meeting the trainer reads out loud a visualization exercise. Participants carry out the exercise, which may also encourages them to envision themselves as a non-smoker. |
| B8. | Nicotine replacement therapy (NRT) | **A** | - Coping with cravings | - Facilitation | Participants who receive free nicotine patches and tablets (if applicable). |
| B9. | Free tickets for public transport | **N** | - Facilitate participation | - Facilitation | Participants receive 2 public transport tickets in every session: 1 ticket for the way back home and 1 ticket to return to the next session. |
| B10. | Stamp for presence and certificates for participation | **N** | - Increase motivation for continued participation | - Provide contingent rewards | When present, participants receive a stamp in their workbook. After attending 5 sessions they receive a certificate: "Congratulations! You already attended 5 sessions. Keep it up!" Those who attended (almost) 11 sessions and resign receive a certificate signed by the trainer. |

**Supplementary file 2C. Smoking cessation rolling group intervention: Content for each group meeting**

| **No.** | **Theme of the meeting** | **Intervention component** | **Existing (E)**  **Adapted (A)**  **New (N)** | **Objective(s)** | **Theoretical method(s)** | **Practical application(s)** |
| --- | --- | --- | --- | --- | --- | --- |
| C1. | The balance | - Taking stock of smoking and smoking cessation | **E** | - Show participants that the pros of smoking cessation outweigh the pros of smoking | - Belief selection - Discussion | Weighing the pros and cons of smoking and smoking cessation using a matrix with four sections (pros of smoking, cons of smoking, pros of quitting, cons of quitting) and discussing it plenary. |
| C2. | Delicious healthy eating | - Neighborhood professional: dietician or nutritionist | **A** | - Increase knowledge about a healthy food products - Participants get acquainted with the service and are encouraged to participate | - Provide information - Direct experience | The professional gives information about a healthy diet. Subsequently, participants discuss labels of different food products. |
|  |  | - Plenary discussion about prevention of weight gain | **N** | - Increase knowledge about healthy eating - Increase social support | - Discussion - Mobilize social support | Participants tell each other about weight gain experiences due to smoking cessation. They are encouraged to exchange practical solutions to prevent weight gain. |
| C3. | Emotions | - Experiential expert | **N** | - Increase self-efficacy to quit smoking - Learn to cope with emotions | - Modelling - Provide opportunities for social comparison | The experiential expert shares his experiences with smoking cessation and tells that he used smoking to cope with emotions. He explains how he copes with emotions in a different way since he quit smoking. |
|  |  | - Plenary discussion about coping with emotions | **N** | - Learn to cope with emotions | - Mobilize social support - Counterconditioning | The trainer initiates a plenary discussion about coping with emotions and encourages participants to give each other advice about alternative strategies that they can use instead of cigarettes to cope with emotions. |
| C4. | Desires and possibilities | - Neighborhood professional: employee of a community center | **N** | - Get acquainted with meaningful daytime activities that may help to prevent boredom | - Provide information - Active learning | The professional tells about the various activities that are offered within the community center and plays a game about these activities with the participants. |
|  |  | - Plenary discussion about activities | **N** | - Motivate participants to participate in different activities | - Goal setting - Action planning - Mobilize social support | The trainer asks participants what activities they would like to do and discusses with them how they can start with this. |
|  |  | - Alternative activities | **N** | - Participants are provided with activities to substitute smoking - Learn to cope with stress and boredom | - Counterconditioning - Facilitation | Participants can pick out one of the alternative activities that are offered, such as an embroidery, a coloring book, a puzzle book or a rubix cube. |
| C5. | Nice to exercise | - Neighborhood professional: teacher of a physical activity class | **A** | - Get acquainted with an activity that stimulates physical activity - Encourage participants to participate in the activity | - Provide information - Direct experience | The professional explains the importance of physical activity and does a physical activity with the participants, such as dance. |
|  |  | - Plenary discussion about physical activity | **N** | - Motivate participants to increase their physical activity levels | - Mobilize social support - Planning coping responses - Goal setting - Action planning | The trainer asks participants in what physical activities they already engage, if they want to become more active and how they will do this. |
| C6. | Pitfalls and strategies | - Experiential expert | **N** | - Increase self-efficacy to quit smoking - Learn to cope with difficult situations | - Modelling - Provide opportunities for social comparison | The experiential expert shares his experiences with smoking cessation. He explains how he changed his habits and how he coped with difficult situations. |
|  |  | - Coping strategies | **N** | - Offer strategies to learn to cope with cravings | - Planning coping responses - Cue avoidance - Counterconditioning - Reinforcement | The trainer explains 4 different coping strategies that participants can use when they have cravings: avoidance, acting differently, thinking differently and self-rewarding. She asks participants to give examples for each strategy. |
|  |  | - Pitfalls | **N** | - Create awareness about pitfalls - Learn to cope with difficult situations | - Planning coping responses - Mobilize social support | The trainer asks participants what their pitfalls are and how they can cope with them in the future. They stimulate participants to advise each other. |
| C7. | Keep it up! | - Neighborhood professional: pulmonologist | **N** | - Increase knowledge about smoking and smoking cessation | - Active learning - Individualization | The professional performs a quiz with questions about smoking and smoking cessation. Participants are given ample opportunity to ask questions to the pulmonologist. |
|  |  | - Scenario | **N** | - Learn to cope with difficult situations | - Scenario-based risk information - Planning coping responses | The trainer reads out loud a scenario about a woman who relapses and asks participants what 'small decisions' have led to her relapse. Participants are encouraged to say what the woman in the scenario should have done differently. |
| C8. | Thinking different, acting different | - Experiential expert | **N** | - Increase self-efficacy to quit smoking - Increase self-efficacy to achieve goals | - Modelling - Provide opportunities for social comparison | The experiential expert shares his experiences with smoking cessation and explains how he changed his thoughts when he quit smoking. He also tells how this changed his life and what new goals he has achieved since he quit smoking. |
|  |  | - Positive- and negative thought circles | **N** | - Learn to change negative thoughts into positive thoughts in order to change unhealthy habits into healthy habits | - Consciousness raising - Belief selection | Participants learn about the relationship between positive or negative thoughts and following feelings and actions by filling in positive and negative thought circles in their workbook. |
| C9. | Count your gains | - Neighborhood professional: debt counsellor | **N** | - Get acquainted with the service that the professional offers and encourage participation | - Provide information - Active learning - Feedback | The professional tells about the service he provides and how people can apply for it. She calculates with the participants how much money they save when/since they quit smoking. |
|  |  | - Plenary discussion about the gains of smoking cessation | **A** | - Create awareness about the advantages of smoking cessation | - Consciousness raising - Feedback | Participants are asked what gains they made or expect to make in health and mentally after quitting smoking. The trainer stimulates participants to give each other positive feedback. |
| C10. | Difficult, difficult | - Experiential expert | **N** | - Increase self-efficacy to quit smoking | - Modelling - Provide opportunities for social comparison - Belief selection | The experiential expert shares his experiences with smoking cessation and tells about the gains he made by quitting smoking. |
|  |  | - Discussing withdrawal symptoms and coping | **E** | - Increase knowledge - Learn to cope with withdrawal symptoms | - Provide information - Planning coping responses - Mobilize social support | The trainer gives information about withdrawal symptoms and gives advice about methods to cope with it. The trainer also stimulates participants to advice each other about coping responses. |
|  |  | - Assignment “Proud” | **E** | - Increase self-image - Increase self-efficacy to quit smoking | - Looking back at past successes - Self-affirmation task | In this assignment participants are asked to indicate what situation in the past they are proud of, what they thought about themselves, how they felt and how they can use this sense of pride to quit smoking. |
|  |  | - Short movie about the health consequences of smoking | **E** | - Create awareness about harmfulness of smoking | - Consciousness raising - Increasing knowledge - Persuasive communication | A short movie is shown that provides information about the health consequences of smoking. |
| C11. | Relax | - Neighborhood professional: trainer of a relaxing activity | **N** | - Learn to cope with stress | - Direct experience - Stress management | The professional tells about the importance of relaxation and performs a relaxing exercise with the participants. |
|  |  | - Plenary discussion about coping with stress | **A** | - Learn to cope with stress | - Planning coping responses - Mobilize social support | The trainer discusses with participants what stress they experience and how they can cope with it. The trainer encourages participants to give each other advice. |
